# Intake of food groups and cervical cancer in women at risk for cervical cancer: A nested case-control study

**DOI:** 10.22088/cjim.13.3.599

**Published:** 2022

**Authors:** Mogge Hajiesmaeil, Samaneh Mirzaei Dahka, Ruin Khorrami, Samira Rastgoo, Fatemeh Bourbour, Sayed Hossein Davoodi, Fatemeh Shafiee, Maryam Gholamalizadeh, Saheb Abbas Torki, Mohammad Esmail Akbari, Saeid Doaei

**Affiliations:** 1 Department of Biology and Biotechnology "Charles Darwin", Sapienza University of Rome, Rome, Italy.; 2 Student Research Committee, Guilan university of Medical Sciences, Rasht, Iran; 3 Garmsar Islamic Azad university. Garmsar, Iran.; 4 Departments of Clinical Nutrition and Dietetics, Faculty of Nutrition and Food Technology, National Nutrition and Food Technology Research Institute, Shahid Beheshti University of Medical Sciences, Tehran, Iran.; 5 Faculty of Nutrition and Food Technology, Departments of Clinical Nutrition and Dietetics, National Nutrition and Food Technology Research Institute, Shahid Beheshti University of Medical Sciences, Tehran, Iran.; 6 Nutrition Research Center, School of Nutrition and Food Sciences, Shiraz University of Medical Sciences, Shiraz, Iran.; 7 Student Research Committee, Cancer Research Center, Shahid Beheshti University of Medical Sciences, Tehran, Iran.; 8 Department of Nutrition, Faculty of Nutrition Sciences, Shiraz University of Medical Sciences, Shiraz, Iran.; 9 Cancer Research Center, Shahid Beheshti University of Medical Sciences, Tehran, Iran.; 10 Reproductive Health Research Center, Department of Obstetrics and Gynecology, Al-Zahra Hospital, School of Medicine, Guilan University of Medical Sciences, Rasht, Iran

**Keywords:** Cervical cancer, Cervical intraepithelial neoplasia, Dietary intake, Food group.

## Abstract

**Background:**

The risk of cervical cancer was reported to be influenced by dietary components. This study aimed to illustrate the association between cervical cancer with the intake of food groups in women with a history of cervical neoplasia.

**Methods:**

This nested case-control study was conducted in 558 people with a history of cervical intraepithelial neoplasia (CIN), including 279 women with cervical cancers and 279 controls with low-grade squamous intraepithelial lesions (LSIL). A validated food frequency questionnaire (FFQ) was used to assess the intake of food groups.

**Results:**

The intake of fruits and vegetables in the case group was significantly lower than the control group (P=0.001). Low intake of dairy products, vegetables, and fruits was associated with cervical cancer risk (OR=4.67; 95% CI 1.2-9.49, P=0.001; OR=9.75, 95% CI 1.36-19. 51, P=0.001; and OR=4.82, 95% CI 1.09-7.25, P=0.001, respectively). After adjusting for age, family history, age at first menstruation, number of children, history of vaginal infection, and age at first sexual intercourse, the results were still significant. Additional adjustments to BMI did not change the results.

**Conclusion:**

The results indicate that the risk of cervical cancer can be affected by the intake of certain food groups. Further longitudinal studies are needed to confirm these findings and determine the underlying mechanism of the influence of dietary components on cervical cancer risk.

Cervical cancer is one of the most prevalent types of cancer in women worldwide. It is the fourth most commonly diagnosed cancer (1-3) and the fourth leading cause of cancer death in women. High prevalence of cervical cancer in developing countries (4) may be due to changes in the related factors such as lifestyle (5). The high prevalence and also high cost of treatment of cervical cancer lead to the enormous economic burden (6). Cervical cancer usually develops after a prolonged stage of precancerous cervical lesions (7, 8). Identifying factors related to cervical lesions is essential to reduce the incidence and mortality of cervical cancer. Human papillomavirus (HPV) infection has a key role in the development of cervical cancer (1, 9, 10). Other related factors included multiple sexual partners, using oral contraceptive pills (OCPs) for more than five years, early start of sexual life, multiple pregnancies, unhealthy diet, and smoking (11-14). Many lifestyle factors could influence on cervical cancer (3, 15). According to the studies on cervical cancer, cancer prevention can be achieved by improving the host's immune system through adopting a balanced diet (16-18). 

Some nutrients such as vitamins, minerals, dietary fiber, plant sterols, carotenoids, and various phytochemicals may help prevent multiple cancers through their antitumor, antioxidant, and anti-inflammatory activities (19-22). Moreover, recent experimental evidence has indicated that vitamin and mineral deficiencies are associated with reduced immune function, higher DNA damage, and a higher risk of cervical cancer (22, 23).

Several studies reported that consuming plant-based foods (18), fruits and vegetables (24-26), and particularly cruciferous vegetables (27), vitamin C (28-30), carotenoids (29, 30), vitamin E (26, 29), lycopene (26), folate (31, 32), and vitamin A (30, 33) are associated with a reduced risk of cervical cancer. However, no association was found between the risk of cervical cancer and foods and nutrients intake in several studies (34-36). 

In addition, the association between the food intake and the risk of cervical cancer in people with a history of cervical neoplasia has not been investigated. Dietary intake can effectively exacerbate or improve cervical problems. Therefore, this case-control study aims to determine the association between cervical cancer and dietary intake in Iranian women.

## Methods

This nested case-control study was conducted in 2018 in women with a history of cervical intraepithelial neoplasia II (CIN II) in the last 5 years at the pathology department of Mirzakoochakkhan Gynecology Hospital in Tehran, Iran. All patients (n=558) were followed up with physical examination every 3 months for 5 years. 

The inclusion criteria included the age range between 20 to 45 years, a pathologically confirmed history of CIN, no smoking, no contraceptive pills for more than five years, no immune system disorders, and receiving similar treatment during the last 5 years. The case group included 279 women with the latest histological diagnosis of high-grade CIN III and invasive cervical cancer (ICC) (aged 25–53 years, mean 39±7.04). 

The control group consisted of 279 people with a low-grade lesion (CIN I) after five years of treatment (aged 26–54 years, mean 33.37±6.4). Data on demographic factors, weight, height, body mass index (BMI), and medical history were collected from patients' records.

**Dietary intake: **A validated food frequency questionnaire (FFQ) (37)was used to assess participants' food intake in the last year before cervical cancer diagnosis, including cereals, protein foods, dairy products, vegetables, and fruits through face-to-face interviews. The collected data were classified as low intake (below the recommended minimum serving size) and adequate intake (exceeding the recommended minimum serving size) according to My Plate recommendations of the United States Department of Agriculture (USDA).

**Statistical analysis: **Independent sample t-test and chi-square test were used to compare the general characteristics and dietary intake between two groups. Logistic regression method was used to compare dietary intake among the case and control groups as crude (model 1), after adjustment for family history, first menstrual age, child number, vaginal infection history, first intercourse age (model 2), and after further adjustment for BMI (model 3). All analyses were done using SPSS Version 21 (p<0.05).

## Results

The cases had a higher BMI (27.32 kg / m2 ± 4.69 vs 24.67 kg / m2 ± 2.79, P=0.01) and a higher family history of cervical cancer (21% vs 13%, P=0.04) compared with the control group. There was no significant contrast between the case and the control groups in terms of age, number of children, age of first intercourse, and age of first infection (table 1).

The fruit and vegetable intake of the case group was significantly lower than the control group (52.7% vs 90.7%, P=0.001 and 8.6% vs 98.9%, P=0.001, respectively). Logistic regression on comparison of the dietary intake between the groups (table 3) identified that the lower intake of dairy (< 2 servings/d), vegetables (<4 servings/d), and fruits (< 4 servings/d) were associated with cervical cancer risk (OR=4.67, p= 0.001; OR= 9.75, P= 0.001; and OR= 4.82, P= 0.001, respectively). 

There was no association between the intake of grains and protein foods and the risk of cervical cancer (model 1). The magnitude of the risk increased for low intake of dairy, vegetables, and fruits after adjusting for age, family history, first menstrual age, child number, vaginal infection history, first intercourse age, and using UCPs (OR=5.85, P = 0.001; OR= 12.01, P= 0.001; and OR= 6.18, P = 0.001, respectively) (model 2). Further adjusted for BMI did not change the results (model 3).

**Table 1:** Characteristics of the case group and the control group

	**Cases (n=279)**	**Controls (n=279)**	P*
Age	32.5 (±1.98)	32.34 (±2.2)	0.38
Height	162.49 (±5.19)	161.10 (±96)	0.11
Weight	72.05 (±12.02)	64.01 (±7.71)	0.01
First intercourse age	20.78 (±2.84)	20.31 (±3.13)	0.07
Child number	2.08 (±1.29)	2.14 (±1.25)	0.55
Age of first infection	20.78 (±2.84)	20.31 (±3.13)	0.07
BMI	27.32 (±4.69)	24.67 (±2.79)	0.01
Family history			
Yes	58 (21%)	36 (13%)	0.04
No	214 (77%)	239 (86%)	

^ *^Independent sample t-test and Qi-squared test; BMI: body mass index

**Table 2:** Participant's dietary intake

	**Cases (n=279)**	**Controls (n=279)**	**P** ^*^
**Grains**			
< 6 servings	109 ( 39.1)	105 (37.6)	0.397
≥ 6 servings	170 (60.9%)	174 (62.4)	
**Protein foods**			
< 3 servings	98 (35.1%)	97 (34.8%)	0.5
≥ 3 servings	181 (64.9%)	182 (65.2%)	
**Dairies**			
< 2 servings	59 (21.1%)	52 (18.6)	0.262
≥ 2 servings	220 (78.9%)	227 (81.4%)	
**Fruits**			
< 4 servings	132 (47.3%)	26 (9.3%)	0.001
≥ 4 servings	147 (52.7%)	253 (90.7%)	
**Vegetables**			
< 4 servings	255 (91.4%)	3 (1.1%)	0.001
≥ 4 servings	24 (8.6%)	276 (98.9%)	

^ *^Independent sample t-test

**Table 3:** Logistic regression of the association of dietary intake and cervical cancer

	**Model 1**			**Model 2**			**Model 3**		
	**OR**	**CI 95%**	**P**	**OR**	**CI 95%**	**P**	**OR**	**CI 95%**	**P**
Grains	0.27	0.03-1.9	0.57	0.48	0.23-1.6	0.330	0.06	0.02-1.2	0.54
Protein foods	0.12	0.01-1.3	0.81	0.08	0.04-0.51	0.88	0.05	0.04-0.38	0.93
Dairy	4.67	1.2-9.49	0.001	5.85	1.34-12.83	0.001	4.76	1.32-9.18	0.001
Vegetables	9.75	1.36-19.51	0.001	12.01	5.67-15.14	0.001	12.55	8.25-14.67	0.001
Fruits	4.82	1.09-7.25	0.001	6.18	1.21-9.24	0.001	5.77	1.59-9.64	0.001

Model 1: Crude model 2. Adjusted according to age, family history, first menstrual age, child number, vaginal infection history, first intercourse age.

Model 3. Further adjustment for BMI

## Discussion

This study identified that the patients with cervical cancer had higher BMI and lower intake of dairy, fruits, and vegetables. Adjustments for age, family history, first menstrual age, number of deliveries, history of vaginal infection, age of first intercourse, and using UCPs did not change the significant results. Consistent with this study, the direct relationship between cervical cancer risk and insufficient fruit and vegetable intake has been reported (24, 38). Hwang et al. (24) reported the effect of eating fruits and vegetables in reducing cervical cancer risk (OR = 2.84, 95% CI 1.26- 6.42, P= 0.06 for vegetables; OR = 2, 93, 95% CI 1.25-6. 87, P= 0.01 for fruits). In addition, Vereault et al.(38) showed that dark green or yellow vegetables was related with lowered cervical cancer (RR = 0.4, 95% CI 0.3-1.1, dark green p <0.01 or yellow vegetables; RR = 0.3, 95% CI 0.2-0.6, juice p<0.01). Furthermore, Tomita et al. (39) reported that the increased intake of α and γ tocopherols and increased intake of leafy vegetables and yellow vegetables and fruits were associated with a 50% reduction in CIN3 risk (OR=0.26, CI=95% 0.15-0.47, p<0.001 for α tocopherol; OR=0.46, CI=95% 0.29-0.73, p<0.001 for γ tocopherols; OR=0.46, CI=95% 0.31-0.70, p<0.001 for dark leafy vegetables and yellow vegetables and fruits). Barany et al.(40) reported that the frequency of vegetables and legumes among cervical cancer patients was lower than the others. The intake of vitamins C, D, and folate was considerably lower compared with the control group at the time of diagnosis. In addition, the result of another study by Herrero et al.(41) supported the protective effects of vitamin C (OR= 0.59, CI=95% 0.5-0.9, p<0.0001), carotenoids (OR= 0.60, CI=95% 0.4-0.9, p<0.0001), and other nutrients in fruits and vegetables against the development of invasive cervical cancer.

Chaitali et al. (42) emphasized that fruits and vegetables, dietary fiber (OR= 0.57, CI=95% 0.38-0.87, P= 0.03), vitamin C (OR=0.69, CI=95% 0.47-1.03, P=0.02), vitamins E (OR=0.58, CI=95% 0.38-0.88, P=0.002), vitamin A (OR=0.66, CI=95% 0.45-0.98, P=0.04), α-carotene (OR=0.68, CI=95% 0.46-0.98, P=0.02), folate, lutein (OR=0.76, CI=95% 0.52-1.10, P=0.02), and lycopene (OR=0.81, CI95% 0.57-1.15, P=0.16) may play an essential role in decreasing the cervical cancer risk, independent of other non-nutritional factors. Gonzalez et al. (43) performed the first cohort study to examine the association between fruits and vegetables with the incidence invasive squamous cervical (ISC) and found a significant inverse relationship between fruits (Hazard Ratio (HR)=0.83; CI95% 0.72–0.98) and green and dark leafy vegetables (HR= 0.88; CI95% 0.70-1.10) consumption and ISC risk. 

On the other hand, the results of some studies were in contrast with the findings of the present study. For example, Regina et al.(35) found no reduction in the risk of invasive cervical cancer by increasing intake of dark vegetables (OR=1.33, CI95% 2.5-4.0, P= 0.69), yellow and orange sausages (OR=1.14, CI95% 0.55-1.2, P=0.32), fruits (OR=0.93, CI95% 7.4-12, P=0.26), or legumes (OR=1.02, CI95% 2.9-4.6, P=0.36). In another study, Labani et al. (44) reported that dietary group intake did not differ significantly between cervical cancer patients and normal individuals. Since the patients included in this study were from low-income and poor social classes, it was observed that the overall consumption of food, especially fruits and vegetables was low in both the case and control groups, and there was no clue about the role of diet in the development of cervical cancer. 

The exact mechanism of the influence of fruits and vegetables intake on the risk of cervical cancer has not yet been determined. Some nutrients in fruits and vegetables may play a role in cervical cancer prevention, including antioxidants such as vitamin C (45), vitamin A (46), folate (32), lycopene (39), and vitamin E (39), and dietary fiber (24). The level of oxidative stress is involved in the pathogenesis of cervical cancer, so antioxidant deficiency can be an essential factor in cervical cancer (47). Several studies reported that taking antioxidant vitamins supplements decreased the risk of cervical cancer (30, 48). 

Free radicals from reactive oxygen species form a natural cascade reaction leading to phosphorylation of activator protein 1 (AP1), which is a transcription factor responsible for the expression of a variety of genes that can alter cell growth and apoptosis, such as the carcinogenicity of HPV E6 and E7 Protein. (24, 49, 50). In addition, food ingredients, including antioxidants in fruits and vegetables, can reduce viral load and shorten the duration and progression of disease. The balance of oxidants and antioxidants is also very important in body immune function. It affects the preservation of immune cell membrane lipids and controls the signal transduction and gene expression of immune cells. These may be influence on the risk of HPV infection (24, 51, 52).

Evidence suggests that micronutrient deficiencies may contribute to DNA damage and can lead to tumorigenesis (23). Vitamin A regulates cell growth and differentiation by activating gene transcription via retinoic acid receptors (RAR) and α, β, and γ retinoid X receptors (RXR_s_) (53, 54). The effect of retinoids is most likely exerted at the stage of tumor development. Retinoids inhibit tumor progression by inhibiting proliferation and inducing apoptosis (55, 56). Moreover, vitamin C acts as a potent reducing agent in several hydroxyl reactions and reduces compounds such as oxygen and nitrate. It also prevents malignant deformation and reduces chromosomal damage in cells. Lycopene, a pigment found in fruits and vegetables, acts as a powerful antioxidant and prevents DNA damage by protecting 2-deoxyguanosine from free oxygen damage. It suppresses insulin-like growth factor-stimulated cell proliferation. The American Cancer Society has described the various effects of lycopene such as reduced tumor size (57). The protective effect of α-tocopherol may be mediated by its effect against HPV, which is strongly involved in the etiology of cervical cancer (58).

On the other hand, some studies have shown that nutrients can affect the expression of cancer-related genes and also play a key role in cancer risk in people who are genetically predisposed (59, 60). In addition, people who carry polymorphisms in genes encoding enzymes involved in cellular metabolism may need to different levels of nutrients to prevent cancer (61, 62). In the present study, both case and control groups had a history of intraepithelial neoplasia, this means that both groups were at risk of cervical cancer. The results of this study show that the intake of dairy products, fruits and vegetables reduces the risk of cervical cancer in people who are susceptible to cancer. This may be partly due to the role of nutrients in the development of genetic predisposition to cervical cancer. Further studies on the interaction of nutrition and genetics in patients with cervical cancer are recommended.

The strength of this study was that the participants all had a history of cervical neoplasia. The results of this study can well explain the role of food intake in the development of cervical problems. However, the present study has some limitations. The results could be influenced by self-report dietary measurement method, which is a common limitation in the epidemiological studies (63). Another limitation of this study is the lack of reliable information on HPV status. (42). Future research should examine nutrient-independent effects on cervical cancer along with accurate HPV status measurements. In addition, risk factors for HPV infection or ancillary factors that affect the progression of HPV infection to intraepithelial lesions or cancer must be determined. Also, consider accurately monitoring micronutrient intake and checking their serum levels is recommended (42).

This study confirmed that the risk of cervical cancer could be affected by several aspects of the diet. The low intake of dairy products, fruits and vegetables was associated with an increased risk of cervical cancer. Check these findings and require more longitudinal studies to identify the mechanisms of eating dietetic intake on the risk of female cervical cancer. 
